# 

**Published:** 1935

**Authors:** 


### The Medical Library of the University of Bristol,

WITH WHICH IS INCORPORATED

**The Library of the Bristol Medico-Chirurgical Society.**

*The following donations have been received since the publication of the list in September*, 1935.

December, 1935.

| | |
|---|---|
| Professor R. J. Brocklehurst (1) .. .. | 1 volume. |
| Buenos Aires University (2) .. .. .. | 1 ,, |
| Dr. A. L. Flemming (3) .. .. .. .. | 1 ,, |
| Professor E. W. Hey Groves (4) .. .. | 4 volumes. |
| Dr. M. G. Hughes (5) .. .. .. .. | 1 volume. |
| B. M. Jenkins, Esq. (6) .. .. .. .. | 4 volumes. |
| Dr. Marion S. Linton (7).. .. .. .. | 14 ,, |
| Michell Clarke Memorial Fund (8) .. .. | 21 ,, |
| Munro Smith Memorial Fund (9) .. .. | 6 ,, |
| Professor J. A. Nixon (10) .. .. .. | 5 ,, |
| Peking Union Medical College (11) .. .. | 1 volume. |
| Professor C. Bruce Perry (12) .. .. .. | 1 ,, |
| Professor A. Rendle Short (13) .. .. .. | 1 ,, |
| Smithsonian Institute of Washington (14) .. | 1 ,, |
| Dr. J. Odery Symes (15) .. .. .. | 3 volumes. |
| Wellcome Research Institution (16) .. .. | 1 volume. |

Unbound periodicals have also been received from Dr. J. V. Blachford, Professor E. W. Hey Groves, Professor C. Bruce Perry, and Dr. J. Odery Symes.

### THE ONE HUNDRED AND FIFTY-THIRD LIST OF BOOKS.

The figures in round brackets refer to the figures after the names of the donors. The books to which no such figures are attached have either been bought from the Library Fund or received through the *Journal.*

**Aitken, D. McCrae** .. *Hugh Owen Thomas; his Principles and Practice* .. .. .. .. .. .. .. .. 1935

**Arana, G. B.** .. .. *Sincronización Quirúrgica. Team Standard Operatorio Sincronizado Apendicectomia* (2) 1934

**Ashford, B. K.** .. .. *A Soldier in Science . . . the Autobiography of B. K. Ashford* .. .. .. .. .. .. 1934

**Bailey, H.** .. .. .. *Physical Signs in Clinical Surgery* .. 5th Ed. 1935

**Banister, H.** .. .. *Psychology and Health* .. .. .. .. (8) 1935

**Barcroft, J.** .. .. *Features in the Architecture of Physiological Function* .. .. .. .. .. .. .. (9) 1934

**Beesly, L., and Johnston, T. B.** *Manual of Surgical Anatomy.* Revised by John Bruce and Robert Walmsley 4th Ed. 1935

*Biblioteca Clásica de la Medicina Española.* 4 volumes .. ..(10) 1923

**Bigger, J. W.** .. .. *Handbook of Bacteriology* .. .. 4th Ed. 1935

**Blacker, C. P.** .. .. *Voluntary Sterilization* .. .. .. .. (8) 1934

**Brain, W. R., and Strauss, E. B.** *Recent Advances in Neurology* 3rd Ed. (8) 1934

**Brend, W. A** .. .. *Health and the State* .. .. .. .. (7) 1917

*British Pharmaceutical Codex, The* .. .. .. .. .. .. .. .. .. (8) 1934

**Broderick, F. W.** .. *Pyorrhœa Alveolaris* .. .. .. .. (8) 1931

**Burt, C.** .. .. .. *The Subnormal Mind* .. .. .. .. (15) 1935

**Campbell, A., and Poulton, E. P.** *Oxygen and Carbon Dioxide Therapy* 1934

**Colyer, J. F., and Sprawson, E.** *Dental Surgery and Pathology* 6th Ed. (8) 1931

**Cory, R.** .. .. .. *Phrenicectomy* .. .. .. .. .. .. .. (13) [1935]

*Discursos Leidos en la Solemne Sesion Celebrada en la Real Academia Nacional de Medicina . . . para Conmemora el Centenario de la Muerte de Eduardo Jenner* .. .. .. .. .. .. .. .. .. .. (10) 1923

**Donaldson, M.** .. .. *Radiotherapy in the Diseases of Women* .. (8) 1933

**Eden, T. W., and Lockyer, C.** *Gynæcology* .. .. .. 4th Ed. (8) 1935

**Elliot, R. H.** .. .. *A Treatise on Glaucoma* .. 2nd Ed. (8) 1922

**Emmart, E. W.** .. *Concerning the Badianus MSS., an Aztec Herbal "Codex Barberini Latin* 241" (Vatican Library) .. .. .. .. .. .. .. (14) 1935

**FitzGerald, C. T., jun.** *The "Albatross," being a Biography of Conrad FitzGerald, M.R.C.S., L.S.A.,* 1847–1933 [1935]

**Fox, R. F., and Van Breeman.** *Chronic Rheumatism* .. .. (8) 1934

**Frank, L.** .. .. .. *Die Psychokathartische Behandlung Nervöser Störungen* .. .. .. .. .. .. .. (15) 1927

**Friend, G. E.** .. .. *The Schoolboy* .. .. .. .. .. .. .. (8) 1935

**Gabell, D.** .. .. .. *Prosthetic Dentistry* .. .. .. .. .. (8) 1921

**Griges, R.** .. .. .. *Schistosomiasis (Bilharziasis)* .. .. (8) 1934

**Greenwood, A. Y.** .. *Epidemics and Crowd-diseases* .. .. .. 1935

**Gullan, M. A.** .. .. *Theory and Practice of Nursing* .. 4th Ed. 1935

**Gunn, J. A.** .. .. *An Introduction to Pharmacology and Therapeutics* .. .. .. .. .. 4th Ed. 1934
**Hadfield, G., and Garrod, L. P.** *Recent Advances in Pathology* 2nd Ed. 1934
**Hemming, J.** .. .. *The History and Chemical Analysis of the Mineral Water lately discovered in the City of Gloucester* .. .. .. .. .. .. .. .. 1789
**Herd, H.** .. .. .. *The Diagnosis of Mental Deficiency* .. (8) 1930
**Hunt, A.** .. .. .. *Reminiscences* .. .. .. .. .. .. .. .. (4) 1935
**Jacobi, A.** .. .. .. *Therapeutics of Infancy and Childhood* 3rd Ed. (7) 1903
**Kellicott, W. E.** .. *A Textbook of General Embryology* .. (7) 1914
**Kerr, J. M., and others** *Combined Textbook of Obstetrics and Gynæcology* .. .. .. .. .. 2nd Ed. (9) 1933
**Kersley, G. D.** .. .. *The Rheumatic Diseases* .. .. .. .. .. .. 1934
**Kuno, Y.** .. .. .. *The Physiology of Human Perspiration* .. (9) 1934
**Lawrence, R. D.** .. *The Diabetic Life* .. .. .. .. 8th Ed. (8) 1934
**Leriche, R.** .. .. *Angina Pectoris and its Surgical Treatment* 1935
**Lewis, Sir T.** .. .. *Clinical Science* .. .. .. .. .. .. .. (8) 1934
**Livingstone, J. L.** .. *Aids to Medicine* .. .. .. .. .. .. (12) 1935
**Lockhart-Mummery, P.** *Diseases of the Rectum and Colon* 2nd Ed. (9) 1934
**Macdonald, D. M.** .. *The Student's Pocket Prescriber* .. 10th Ed. 1934
**Macleod, J. J. R.** .. *Physiology in Modern Medicine* 7th Ed. (9) 1935
**Marie, J.** .. .. .. *English, French, Italian Medical Vocabulary*
**Marshall, C. R.**.. .. *A Manual of Prescribing* .. .. .. (7) 1908
**Mellanby, E.** .. .. *Nutrition and Disease* .. .. .. .. .. (8) 1934
*Middlesex Hospital Medical School Collected Clinical Papers* .. (4)1934–5
**Moncrieff, A., ed.** .. *A Textbook on the Nursing and Diseases of Sick Children* .. .. .. .. .. 2nd Ed. 1935
**Moor, C. G., and Partridge, W.** *Aids to the Analysis of Food and Drugs* .. .. .. .. .. .. .. .. 5th Ed. 1934
**Morison, R., and Saint, C. F. M.** *An Introduction to Surgery* 3rd Ed. 1935
**Neame, H.** .. .. *Atlas of External Diseases of the Eye* .. 1934
**Nichols, R. H., and Wray, F. A.** *The History of the Foundling Hospital* .. .. .. .. .. .. .. .. .. .. 1935
**Nosworthy, M. D.** .. *The Theory and Practice of Anæsthesia* .. [1935]
**Oliver, J. H.** .. .. *Diabetes Mellitus : a Clinical Study* .. .. 1935
**Osler, Sir W.** .. .. *Principles and Practice of Medicine.* 12th Ed. revision by T. McCrae .. .. .. (9) 1935
**Parsons, Sir J. H.** .. *Diseases of the Eye* .. .. .. 7th Ed. (8) 1934
**Pfaundler, M. and Schlossman, eds.** *The Diseases of Children.* 4 Vols. English translation, edited by H. L. Shaw and L. la Fetra .. .. .. .. .. .. (7) 1908
**Roberts, E.** .. .. *Vegetable Materia Medica of India and Ceylon* .. .. .. .. .. .. .. .. .. (4) 1931
**Robertson, A. W.** .. *Studies in Electropathology* .. .. .. (7) 1918
**Rogers, Sir L., and Megan, Sir J. W.** *Tropical Medicine* 2nd Ed. (8) 1935
**Ross, J. S., and Fairlie, H. P.** *Handbook of Anæsthetics* .. 4th Ed. (3) 1935
**Scott, S. G.** .. .. *Radiological Atlas of Chronic Rheumatic Arthritis (the hand)* .. .. .. .. .. (8) 1935
**Sears, W. G.** .. .. *Medicine for Nurses* .. .. .. .. .. .. .. 1935

| | | |
|---|---|---|
| *Spanish Influence on the Progress of Medical Science, with an Account of the Wellcome Research Institution* .. .. .. .. .. .. .. (16) | | 1935 |
| **Speed, K.** .. .. .. | *A Textbook of Fractures and Dislocations* 3rd Ed. (4) | 1935 |
| **Stephenson, T.** .. .. | *Incompatibility in Prescriptions and how to avoid it* .. .. .. .. .. .. .. 4th Ed. | 1935 |
| **Sutherland, H. G., ed.** | *The Control and Eradication of Tuberculosis* (7) | 1911 |
| **Taylor, A. S.** .. .. | *Principles and Practice of Medical Jurisprudence.* 9th Ed. Edited by S. Smith. 2 Vols. .. .. .. .. .. .. | 1934 |
| **Taylor, J. M., and Wells, W. H.** | *Manual of the Diseases of Children* 2nd Ed. (7) | 1901 |
| **Teacher, J. H.** .. .. | *A Manual of Obstetrical and Gynæcological Pathology* .. .. .. .. .. .. .. .. | 1935 |
| **Tenenbaum, J.** .. .. | *The Riddle of Sex* .. .. .. .. .. .. (5) | 1930 |
| **Ten Teachers** .. .. | *Midwifery* .. .. .. .. .. .. .. 5th Ed. | 1935 |
| **Thomson, H. C.** .. | *The Story of the Middlesex Hospital Medical School* .. .. .. .. .. .. .. .. .. | 1935 |
| **Tubby, A. H.** .. .. | *Appendicitis* .. .. .. .. .. .. .. (7) | 1900 |
| **Tumburus, J.** .. .. | *Sintesis Histórica de la Medicina Argentina* (1) | 1926 |
| **Turner, G. G., ed.** .. | *Modern Operative Surgery.* 2nd Ed. 2 Vols (8) | 1934 |
| **Wakeley, C. P. G.** .. | *Modern Treatment in General Practice.* 2 Vols. | 1935 |
| **Winter, G., and Ruge, C.** | *Textbook of Gynæcological Diagnosis* .. (7) | 1909 |
| **Younger, E. G.** .. | *Insanity in Everyday Practice* .. .. (7) | 1904 |

### TRANSACTIONS, REPORTS, JOURNALS, ETC.

| | |
|---|---|
| Contributions from the Peking Union Medical College, Peking. Vol. III. (11) | 1935 |
| Medical Annual General Index and Review for the ten years 1925 to 1934 .. .. .. .. .. .. .. .. .. .. .. .. .. .. .. .. .. | 1935 |
| Medical and D.P.H. Examination Papers, 1932–35, for the Degrees of the University of Edinburgh, etc. .. .. .. .. .. .. .. .. .. | 1935 |
| Proceedings of the Royal Society of Edinburgh, VI., IX., X., XI... (6) | |
| Prognosis. Vol. I. Published by " The Lancet Ltd.".. .. .. (15) | 1935 |
| St. Thomas's Hospital Reports. Vol. LVII. .. .. .. .. .. .. | 1935 |
| Surgical Clinics of North America. Oct., 1935. Vol. 15, No. 5. " Mayo Clinic " .. .. .. .. .. .. .. .. .. .. .. .. .. | |
| Transactions of the Association of American Physicians. Vol. 50 | 1935 |

---

### Publications Received

From Edward Arnold & Co. :

*The Hair and the Scalp.* By Agnes Savill, M.A., M.D., M.R.C.P.I. Price 12s. 6d.

From John Bale, Sons & Danielsson :

*Slums and Slummers.* By C. R. A. Martin, M.R.S.I., A.M.Inst.M.E. Price 6s.

From CASSELL & CO.:

*An Introduction to General Therapeutics.* By H. K. FRY, M.D., B.Sc., D.P.H. Price 6s.

From HUTCHINSON'S SCIENTIFIC AND TECHNICAL PUBLICATIONS:

*Foundations of Short Wave Therapy.* By WOLFGANG HOLZER, Dr.Ing., and EUGEN WEISSENBERG, Dr. Med. Translated by J. WILSON, F.R.C.P.Ed., D.M.R.E. (Cantab.) and C. M. DOWSE, B.Sc., A.M.I.E.E. Price 12s. 6d.

From H. K. LEWIS & CO. LTD.:

*Urology in General Practice.* By ALEX E. ROCHE, M.A., M.D., M.Ch., F.R.C.S. (Eng.). Price 17s. 6d.

*Chronic Streptococcal Toxæmia and Rheumatism.* By J. D. HINDLEY-SMITH. Price 7s. 6d.

*The Principles and Practice of X-ray Therapy.* By FFRANGCON ROBERTS, M.A., M.D., M.R.C.P., D.M.R.E. Price 10s. 6d.

*Common Skin Diseases.* By A. C. ROXBURGH, M.A., M.D., B.Ch. (Cantab.), F.R.C.P. (Lond.). Third Edition. Price 15s.

*Milk Production and Control.* By WM. CLUNIE HARVEY, M.D., D.P.H., M.R.San.I., and HARRY HILL, M.R.San.I., A.M.I.S.E., M.S.I.A. Price 21s.

*Medical and Other Verses.* By ALEX E. ROCHE. Price 3s. 6d.

From E. & S. LIVINGSTONE:

*A Practical Handbook of Midwifery and Gynæcology.* By W. F. HAULTAIN, B.A., M.B., B.Ch., F.R.C.S. Ed., M.R.C.P. Ed., F.C.O.G., and CLIFFORD KENNEDY, M.B., Ch.B., F.R.C.S. Ed., M.C.O.G. Second Edition. Price 15s.

*Medical and D.P.H. Examination Papers,* 1932–35, *for the Degrees of the University of Edinburgh, and the Diplomas of the R.C.S., R.C.P. Edinburgh, and the Royal Faculty of Physicians and Surgeons, Glasgow.*

*Anatomy* (*Head and Neck*). By C. R. WHITTAKER, F.R.C.S.E., F.R.S.E. Fourth Edition, Part 3. *Histology.* Third Edition. *Physiology.* Fourth Edition, Parts 3–5. "The Catechism Series." Price 1s. 6d. each part.

From OXFORD UNIVERSITY PRESS (HUMPHREY MILFORD):

*The Early Diagnosis of the Acute Abdomen.* By ZACHARY COPE, B.A., M.D., M.S. Lond., F.R.C.S. (Eng.). Price 10s. 6d.

From THE PRESCRIBER EDITORIAL AND PUBLISHING OFFICES:

*Incompatibility in Prescriptions and how to avoid it.* By THOMAS STEPHENSON. Fourth Edition. Price 6s.

From JOHN WRIGHT & SONS LTD.:

*Mentality and the Criminal Law.* By O. C. M. DAVIS, M.D., D.Sc., and F. A. WILSHIRE. Price 5s.

*British Journal of Surgery.* Vol. XXIII. No. 90. Price 12s. 6d.

*Physical Signs in Clinical Surgery.* By HAMILTON BAILEY, F.R.C.S. Fifth Edition. Price 21s.

*An Introduction to Surgery.* By RUTHERFORD MORISON, M.D., F.R.C.S. (Ed.), F.R.C.S. (Eng.), M.A., D.C.L., LL.D., and CHAS. F. M. SAINT, C.B.E., M.D., M.S., F.R.C.S., F.R.A.C.S. Third Edition. Price 15s.

### Local Medical Notes

### BRISTOL EYE HOSPITAL.

The new wing of the Bristol Eye Hospital was declared open on Monday, 21st October, 1935, by Her Grace the Duchess of Beaufort, in the presence of the Lord Mayor of Bristol and about 350 supporters.

The Eye Hospital was founded in 1810, there being at the time only two similar hospitals in the country, namely the Royal London Ophthalmic Hospital, at Moorfields, founded in 1804, and the West of England Eye Infirmary, Exeter, in 1808. As far back as 1886 an idea of an entirely new hospital had been contemplated, but instead further extensions were made to the existing building.

The opening of the new wing provides an up-to-date hospital in place of the converted houses which had previously been in use.

The present building includes a large waiting-hall, out-patients' surgery, and a casualty department: the surgical and treatment wards have some balconies, and there is also a properly equipped children's ward, which had always been urgently required. The kitchens, instead of being in the basement, are now situated on the top of the building with service lifts to each ward kitchen.

### HONOUR.

The King has granted Dr. Gordon Winstanley Spencer authority to wear the Insignia of the Fourth Class (Civil Division) of the Order of Al Rafidain conferred upon him by the King of Iraq in recognition of valuable services rendered by him as Chief Ophthalmic Specialist in the Iraq Health Service.

Dr. Spencer was House Physician at the Royal Infirmary from September, 1913, to April, 1914.

EXAMINATION RESULTS.

**University of Bristol.**—Students of the University have recently passed the following examinations :—

M.D.—*Pass :* N. J. England.

M.B., CH.B.—*Supplementary First Examination. Pass :* Madeline I. Finzel, Gwendolen M. Higgins, H. Mullen, R. D. R. Shanks, D. A. Squire.

M.B., CH.B.—*Final Examination.* (*Section I.*) *Pass :* J. F. Ackroyd (with Distinction in Pathology), Daphne V. Dennis, R. L. J. Derham, M. Dworkis, H. D. T. Gawn, Emily G. Hamlyn, M. E. M. Herford, F. J. W. Hooper, C. R. G. Howard, C. B. Jones, M. M. Lewis, Margaret E. Morgan, R. H. Owen, G. L. Page, A. N. H. Peach, P. J. Ryan (with Distinction in Materia Medica and Pharmacy, Pharmacology and Therapeutics, and Forensic Medicine and Toxicology), J. W. E. Snawdon, A. M. Spencer, Mabel W. N. Tribe.

M.B., CH.B.—*Final Examination. Pass :* V. T. Baxter, Theodora M. Crabtree, J. G. Field, Irene G. Hamilton, M. A. Nicholson.

B.D.S. — *Supplementary First Examination. Pass :* J. R. D. Gordon.

B.D.S.—*Second Examination. Pass :* R. J. Gething.

B.D.S.—*Third Examination. Pass :* F. P. Ewens.

L.D.S. — *Supplementary Preliminary Science. Pass :* K. A. Gordon-Ralph, J. W. S. Jenkins, P. Le Masurier.

L.D.S.—*First Professional Examination. Pass :* H. B. Harding, Peggy M. Rooke, H. D. Williams.

L.D.S.—*Second Professional Examination. Pass :* M. G. Davies, P. H. S. Paine, H. W. Williams.

**Conjoint Board.** — M.R.C.S., L.R.C.P. *Pass : In Physiology :* E. A. Griffiths, R. T. Moore. *In Pharmacology and Materia Medica :* Myrtle M. Baxter, A. L. T. Beddoe, Margaret E. M. Slater, T. R. de V. Vallentin. *In Midwifery :* J. A. Cochrane.

L.D.S., R.C.S.—*Final Examination. Pass : Part I. :* W. White. *Part II. :* R. Edwards, J. G. Jones, G. L. R. Welch.

### Index

American College of Surgeons, Clinical Congress, 132.
Appointments—
J. A. Nixon.—Industrial Health Research Board of the Medical Research Council, 189.
T. B. Davie, Professor of Pathology, University of Bristol, 189.
A. R. Parkes.—Lecturer in Anatomy, University of Bristol, 189.
H. R. Noltie.—Lecturer in Physiology, University of Bristol, 189.
Dorothy Woodman. — Lecturer in Pathology, University of Bristol, 189.
R. G. Paul.—Clinical Lecturer in Surgery, University of Bristol, 189.
H. J. Orr-Ewing.—Clinical Lecturer in Medicine, University of Bristol, 189.
C. H. G. Price.—Assistant Curator of Pathological Museum, 189.
T. S. Hele.—Master of Emmanuel College, Cambridge, 244.
Askins, Robert Arthur—Obituary, 248.

B.M.A. Meeting, Melbourne, 247.
Bristol Eye Hospital—Opening of New Wing, 259.
Bristol General Hospital, Cricket Challenge Cup, 65.
Bristol Royal Hospital for Women and Children—New Nurses' Home, 140.
Bristol Royal Infirmary Nurses' League, 64.
Burgess, N. F. C. — Eczema and dermatitis, 103.

Carleton, H. H.—Habit and illness, 41.
Chambers, E. R.—Nasal sinusitis and infections of the eyeball (résumé), 184.
Colledge, L. — Tracheal obstruction (résumé), 185.
Critchley, M.—Observations on pain (Long Fox Memorial Lecture), 191.

Dermatitis and eczema.—N. F. C. Burgess, 103.

Eczema and dermatitis.—N. F. C. Burgess, 103.

Fitz-Gerald, C., 63.

Foss, Edwin Vincent.—Obituary, 249.

Hudson, B., and Wollaston, F. L.—Clinical Record : a case of pulmonary consolidation of doubtful origin, 233.
Hyperthyroidism, Medical treatment of.—J. I. Noble, 113.

Iles, A. E. — Diseases of lachrymal sac, 151.
Industrial medicine and traumatic surgery, 132.
International Congress of the History of Medicine, Madrid, 244.

Lachrymal obstruction, Treatment of.—J. G. R. Scarff, 159.
Lachrymal sac, Diseases of.—A. E. Iles, 151.
Library, 69, 135, 186, 254.
Long Fox Memorial Lecture. — M. Critchley on Observations on pain, 191.

MacNalty, A. S.—Thomas Marryat—a memoir, 165.
Marryat, T. — A memoir — A. S. MacNalty, 165.

Men-midwives of the past.—M. H. Phillips, 83.
Mental conditions, Results of treatment of.—G. R. de M. Rudolf, 219.
Mollison, W. M.—Operative treatment of vertigo (résumé), 183.
Morris, J.—Characteristics of electrical deaf aids (résumé), 182.

Nixon, J. A.—Licence to practise and liberty to teach medicine in English provinces, 19.
Noble, J. I.—Medical treatment of hyperthyroidism, 113.

Obituaries—
Walter Salisbury, 65.
Henry Waldo, 66.
Edward Robinson, 181.
Robert Arthur Askins, 248.
Edwin Vincent Foss, 249.
Wilfred Leslie Sleight, 250.

Parker, G.—Honorary member of Bristol Medico-Chirurgical Society, 246.
Phillips, M. H.—Men-midwives of the past, 83.
Presidential Address.—Problem of man's origin.—A. R. Short, 1.

Radium Therapists Visiting Club, Bi-annual meeting at Bristol, 251.
Robinson, Edward—Obituary, 181.
Rolleston, Sir H.—Evolution of British orthopædics, 143.
Royal Society of Medicine—
Meeting of Sections of Otology and Laryngology at Bristol University, 182.
Provincial Meeting of Orthopædic Section at Bath, 185.
Rudolf, G. R. de M.—Results of the treatment of mental conditions, 219.

Salisbury, Walter—Obituary, 65.
Scarff, J. G. R. — Treatment of Lachrymal obstruction, 159.
Short, A. R.—Problem of man's origin (Presidential Address), 1.
Silver Jubilee, 130.
Sleight, Wilfred Leslie.—Obituary, 250.

Waldo, Henry—Obituary, 66.
Wollaston, F. L.—See Hudson, B., and Wollaston, F. L.

